# Trends and Non-Clinical Predictors of Respiratory Syncytial Virus (RSV) and Influenza Diagnosis in an Urban Pediatric Population

**DOI:** 10.23937/2469-5769/1510112

**Published:** 2023-03-06

**Authors:** Marina Oktapodas Feiler, Recai Yucel, Zhiqing Liu, Mary Caserta, B. Paige Lawrence, Carter H Pason, Dwight J Hardy, Kelly Thevenet-Morrison, Ann Dozier, Todd A Jusko

**Affiliations:** 1 Department of Epidemiology and Biostatistics, College of Public Health, Temple University, USA; 2 Department of Environmental Sciences, School of Medicine and Dentistry, University of Rochester, USA; 3 Department of Public Health Sciences, School of Medicine and Dentistry, University of Rochester, USA; 4 Department of Pediatrics, School of Medicine and Dentistry, University of Rochester, USA; 5 Department of Microbiology and Immunology, School of Medicine and Dentistry, University of Rochester, USA

**Keywords:** RSV, Flu, Determinants, Risk factors, Predictors

## Abstract

**Objective:**

To evaluate the demographic, maternal, and community-level predictors of pediatric respiratory syncytial virus (RSV) and influenza diagnosis among an urban population of children residing in Rochester, NY.

**Study design:**

A test-negative case-control design was used to investigate various non-clinical determinants of RSV and influenza diagnosis among 1,808 children aged 0–14 years who presented to the University of Rochester Medical Center (URMC) or an affiliated health clinic in Rochester, NY between 2012–2019. These children were all tested for RSV and influenza via polymerase-chain-reaction (PCR) method, including RSV and influenza diagnosis of all severity types. Test results were linked to medical records, birth certificates, questionnaires administered through the Statewide Perinatal Data System, and the US census by census tracts to obtain information on child, maternal, demographic, and socio-economic characteristics.

**Results:**

Overall the strongest predictor of RSV and influenza diagnosis was child’s age, with every year increase in child’s age, risk for RSV decreased (OR: 0.75; 95% CI: 0.71, 0.79) and risk for influenza increased (OR: 1.20; 95%: 1.16, 1.24). In addition to age, non-private insurance type was positively associated with influenza diagnosis. When considering the proportion of positive cases for RSV and influenza over all PCR tests by respiratory season, a spike in influenza cases was observed in 2018–2019.

**Conclusions:**

Age was a strong predictor of RSV and influenza diagnosis among this urban sample of children.

## Introduction

Respiratory syncytial virus (RSV) and influenza are common viral respiratory infections among children. It is estimated that among children under 5 years of age, RSV accounts for 58,000 hospitalizations in the United States and 100–300 deaths, annually [1,2]. RSV is the leading cause of pneumonia and bronchiolitis in children under the age of 1 year in the United States [3]. Approximately 70% of children will have a RSV infection in their first year of life, and of those 50% will have a recurrence in their second year of life [4]. Influenza virus causes annual epidemics and leads to 6,000–26,000 annual influenza-associated hospitalizations among children under 5 years of age, and 600,000–2,500,000 outpatient visits [5]. The influenza virus is vaccine preventable, with annual vaccines needed due to annual differences in circulating strains. Among children, influenza vaccine effectiveness is between 20–60% depending on age, vaccination type, and circulating influenza virus [6].

All pediatric populations are at an increased risk of RSV and influenza infections compared with adults given their naïve immune systems. There is limited literature reporting child, maternal, demographic, and socio-economic predictors of these infections. Most studies have focused on child age, race, ethnicity, daycare attendance, number of siblings in the home, and clinical predictors [7–11]. Preterm birth, low birthweight, maternal smoking, family history of atopy, lack of breastfeeding, and living in an overcrowded household (household with > 7 members) are additional risk factors that have been significantly associated with severe RSV infection among children under five years of age [12]. RSV hospitalizations have been associated with younger child age (< 6 months), male sex, Black race, Hispanic ethnicity, having siblings, and daycare attendance [7–9]. The epidemiologic literature investigating predictors of RSV and influenza diagnoses are heavily targeted to children with severe disease, such as hospitalized children. There is limited evidence on predictors of RSV diagnosis, at all severity levels, despite 2.1 million annual outpatient (non-hospitalization) visits for RSV among children under the age of five [13].

Given the gaps in the literature, a test-negative case-control study was designed to investigate trends of RSV and influenza diagnosis of all severities over 7 respiratory seasons, and a variety of predictors for RSV and influenza diagnosis among an urban sample of children 14 years of age and younger. Our objective was to evaluate child characteristics, maternal characteristics, demographic, and community-level socio-economic (SES) factors as potential determinants of RSV and influenza diagnosis of all severity types.

## Methods

A test-negative case-control study was conducted among children, ≤ 14 years of age, who were tested for RSV and influenza via molecular diagnostic method at URMC, Rochester, NY between October 1, 2012 and May 31, 2019 [14]. This is the gold-standard study design for observational studies to assess infection risk and vaccine effectiveness due to the underlying assumption of equal risk of pathogen exposure among these children [15,16]. We only included RSV and influenza tests performed between October 1^st^ and May 31^st^, in each year to further enhance the underlying assumption of the test-negative study design that both cases and controls have equal risk of pathogen exposure [15]. The typical respiratory season is between October and May when RSV, influenza, and other respiratory viruses circulate more readily, and community exposure is more common. The study was approved by the internal Research Subjects Review Board (RSRB) at URMC (RSRB # 0070915). Informed consent was waived as this was a retrospective, secondary data analysis of existing medical records.

Children clinically tested via real-time polymerase-chain-reaction (PCR) method at the URMC clinical microbiology laboratory were categorized into cases (positive) and controls (negative) based on their test results [14]. Nasopharyngeal swabs were performed on children with suspected respiratory infection based on symptoms, timing of high exposure season, and clinical judgement. For children with multiple respiratory tests and positive results, only the initial positive respiratory test was considered as a case. Children who served as a case and tested negative at a different point from 2012–2019 could also serve as a control. PCR method testing included a panel for influenza A and B, and RSV infection, and a second full respiratory panel include influenza A and B, RSV, and an additional seven respiratory viruses [14]. The URMC laboratory is Clinical Laboratory Improvement Amendments (CLIA)-permitted by the New York State Department of Health, and accredited by the College of American Pathologists to perform PCR method testing for influenza and RSV [14]. Internal quality assurance programs and standard operating procedures have been previously described [14]. From the laboratory, test result, date of testing, and medical record number was obtained.

Data extraction from medical records was done using the Informatics for Integrating Biology and the Bedside (i2b2) system, a clinical data query tool at URMC used to elicit medical record data. Data collected from medical records included child’s date of birth, sex, race, ethnicity, insurance status, address, and zip code. For a subset of children born at Strong Memorial Hospital (SMH) (affiliated with URMC), laboratory and medical records were linked to the Statewide Perinatal Data System (SPDS). SPDS collects information from birth certificates and supplemental questionnaires at nine birthing hospitals in the nine-county Finger Lakes Region since 1998. From SPDS, maternal age, parity, breastfeeding status at delivery, maternal smoking before and during pregnancy, and birthweight were collected. Additional socio-economic variables were obtained by using geographic information system (GIS) and spatial modeling techniques using the ArcGIS software (version 9.2). Children’s addresses at time of PCR test were mapped and geocoded to assess their census tract by using census mapping. These census tracts were then linked to the US census 2010, these census data will be most representatives of socio-economic factors of interest from 2012–2019. Socio-economic (SES) variables included college education (less than bachelor’s degree obtained), living below poverty status, and unemployment. These variables were dichotomized by finding the median proportion of each variable in every census tract included in the study (N = 190), and individuals were coded whether they resided in a census tract where their specific SES variable status was above or below the median proportion for their specific tract.

The full list of predictors considered in the present analysis included child’s age at testing (measured by using date of birth and PCR testing date), sex, race (White, Black, other), ethnicity (Hispanic, non-Hispanic), insurance status (private, public, self-pay), respiratory season of testing (2012–2019), birthweight (grams), maternal age, parity, breastfeeding status during delivery hospitalization (breast milk only, formula only, both breast milk and formula, or other), perinatal smoking (smoking status within 3 months of pregnancy and/or during pregnancy), and community-level unemployment, education, and poverty status. Analyses were performed among children with complete information on all predictors of interest (N = 1,808).

Bivariate analyses examined the crude association between RSV and influenza and all predictors individually using logistic regression to describe the differences between cases and controls among this study sample. Proportions of positive influenza and RSV tests over all PCR tests were examined per age group and respiratory season. Multiple logistic regression models were fit to include all potential predictors for influenza and RSV separately, estimating the adjusted risk of RSV and influenza while considering all other predictors by reporting adjusted odds ratios and 95% confidence limits. Stepwise regression models were used to understand the most important contributors to the risk of RSV and influenza diagnosis. P < 0.010 criterion was used for inclusion into the models. All data analyses were performed using SAS software (version 9.4; SAS Institute Inc.).

We conducted multiple imputations [17] to improve efficiency of the utilization of the data while addressing the uncertainty due to missing data. SAS PROC MI using a sequential regression imputation (SRMI option) was used [18]. Missing data stemmed from the linkage of medical records to SPDS and geo-mapping and US Census linkage. Children included in the study with data from SPDS must have been born at SMH, which to a reduction in our sample with complete information. Additionally, some children could not be mapped within Monroe County in Rochester, NY as they were traveling outside the Rochester region or did not have appropriate addresses (P.O. boxes, misspellings which could not be matched to a known address).

Influenza is a vaccine-preventable disease, considering vaccination as a predictor of influenza diagnosis is ideal. After data extraction and review of the medical records, vaccination status was not recorded consistently. Some children were given a date of vaccination, others had no information. Given the provided data, we were unable to identify whether a child had received a vaccination but was not reported or had not received a vaccination. Among children who had a vaccination reported, an adjusted logistic regression model was fit considering all the potential predictors for influenza.

## Results

Our study sample consisted of 1,808 children between 0 and 14 years of age, with 432 children testing positive for RSV, and 296 children testing positive for influenza. This includes all children tested for RSV and influenza via PCR between October 1-May 31 from 2012 to 2019 at URMC. The study sample reported children who self-identified (by parent/guardian) as 51% White, 36% Black, and 13% other race, and 12% reporting Hispanic ethnicity. When considering bivariate analyses for all predictor variables and RSV and influenza diagnosis, most predictors were positively associated with RSV and influenza, although these results are imprecise and not statistically significant. Compared with controls, RSV cases were younger, non-typical birthweight, male, non-white, not exclusively breastfed at delivery, and had mothers who were multiparous, who smoked before and/or during pregnancy, and resided in an area with a high proportion of less than college education. Compared with controls, influenza cases were older, typical birthweight, non-white race, Hispanic, non-private insurance type, and had younger, multiparous mothers who smoked before and/or during pregnancy.

**Figure 1:**
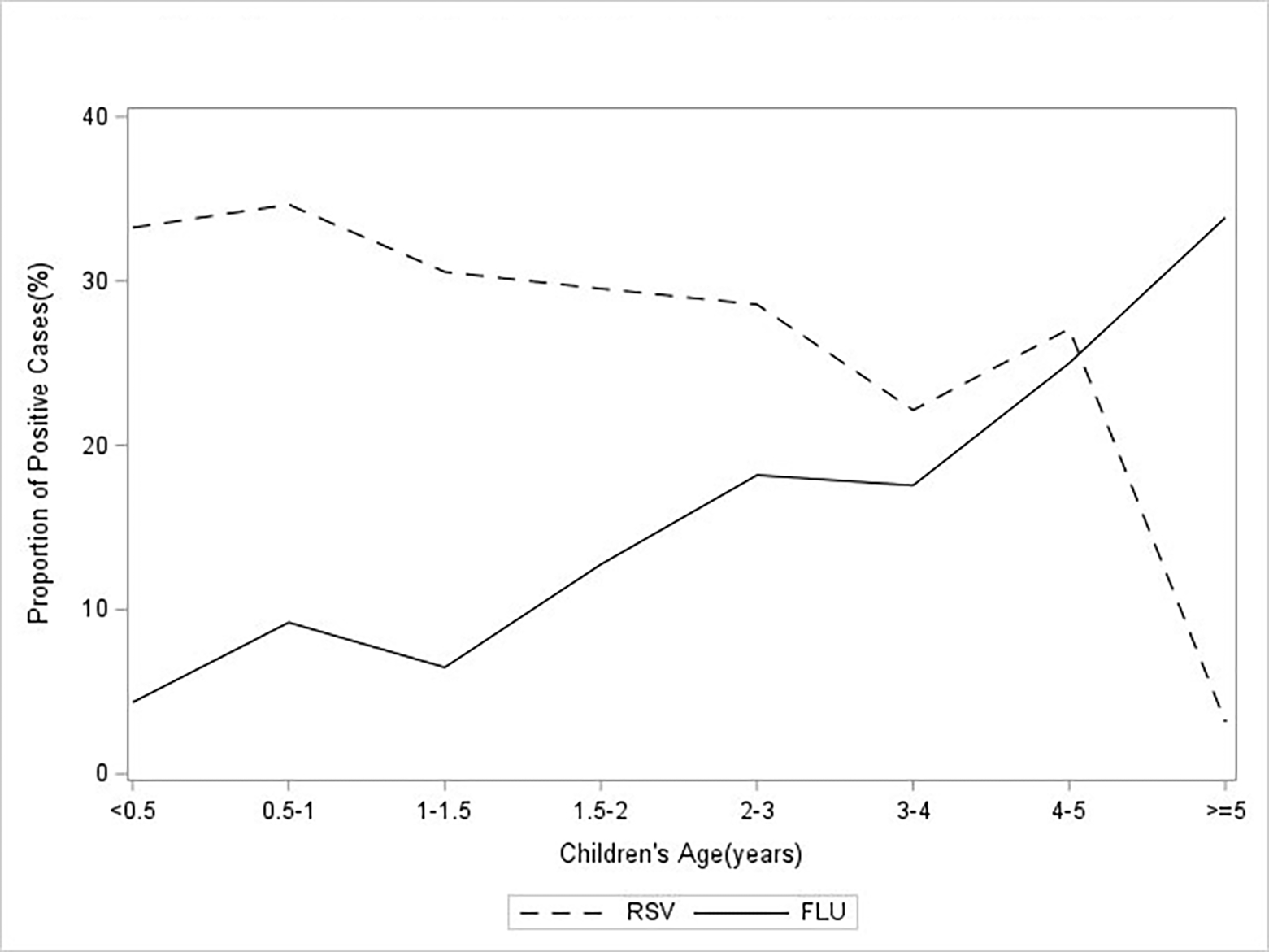
The proportion of positive RSV and influenza PCR tests within each group (N = 1808).

**Figure 2:**
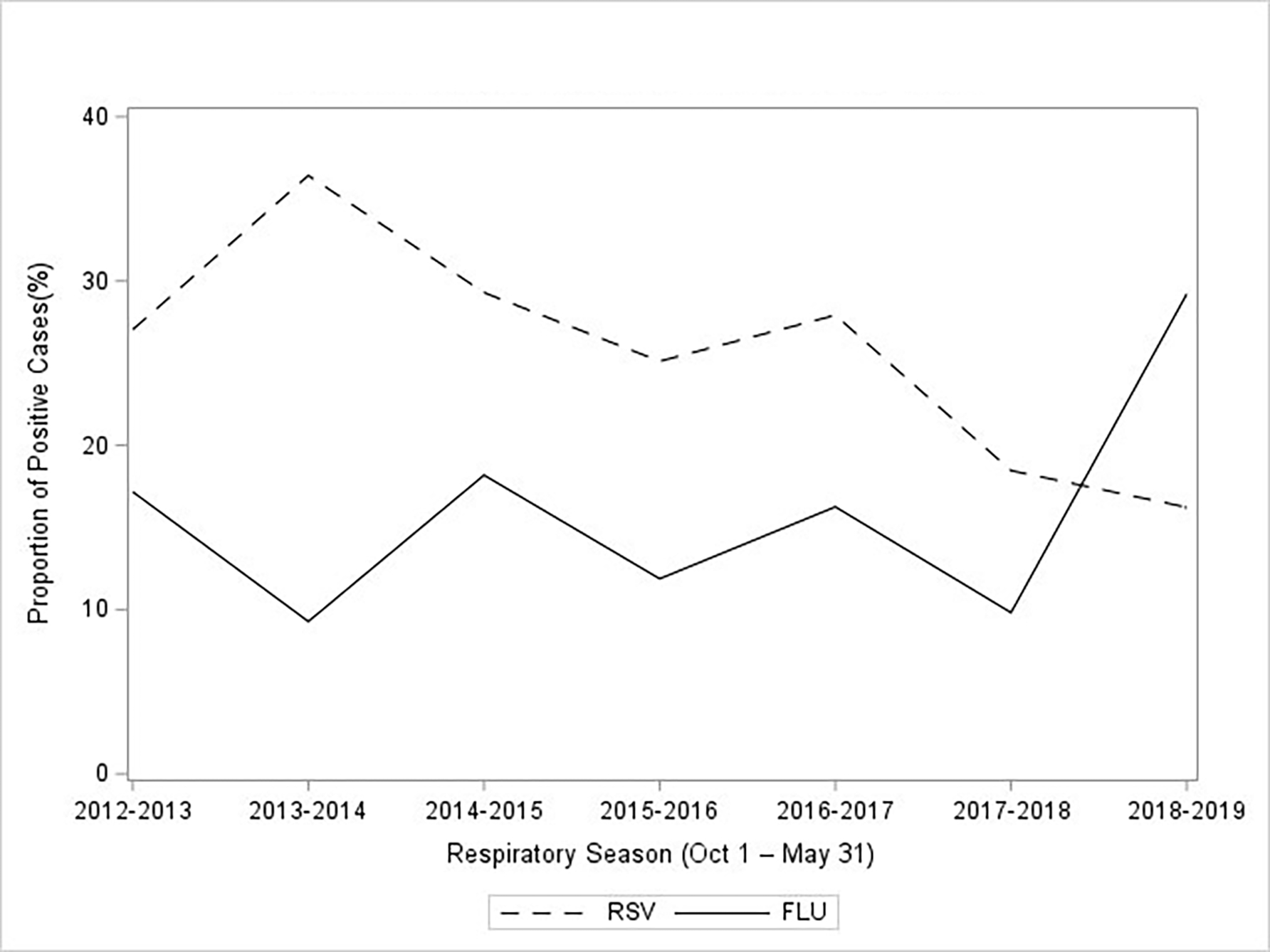
The proportion of positive RSV and influenza PCR tests among all PCR tests per respiratory season, from 2012–2019 (N = 1808).

Median (IQR) age of RSV cases was 1.05 (0.42, 2.06) years, cases ranged from 0.04 years (2-weeks-old) to 11.4 years. For every year increase in age, children had a 15% decreased risk of RSV diagnosis (Table 1), with children under 6 months of age having > 150% increased risk for RSV, 95% CI: 8.61, 27.05 (Table 1). Median (IQR) age for influenza cases was 5.15 (2.22, 7.94) years, cases ranged from 0.12 years (1.5 months) to 14.97 years. In contrast with RSV, for every year increase in age, there was a 20% increased risk for influenza diagnosis, 95% CI: 1.16, 1.24 (Table 1). The proportion of positive RSV tests over all viral PCR tests done in this sample decreased with increasing age (Figure 1). The proportion of positive influenza tests increased with increasing age (Figure 1). When examining the proportion of positive RSV and influenza tests per respiratory season, the 2018–2019 seasons demonstrated the highest proportion of influenza diagnosis and the 2013–2014 seasons demonstrated the highest proportion of RSV diagnosis (Figure 2).

The adjusted multivariable logistic regression model examining risk of RSV diagnosis, observed child’s age to be a strong, statistically significant predictor (Table 2). Given RSV disease is very common in young children, as expected lower child age was associated with an increased risk of RSV diagnosis. After controlling for all other predictors, children younger than 6 months of age had a 14% increased odds of RSV diagnosis compared with children five years of age and older, 14.14 (7.89, 25.56) (Table 2). Birthweight was observed to increase the risk of RSV diagnosis if it was out the typical range of 2,500–4,000 grams. Observed odds ratio for those with a low birthweight (< 2,500 grams) was 1.14 (0.85, 1.52), and for those with a high birthweight (≥ 4,000 grams) was 1.25 (0.79, 1.97) (Table 2). Non-white race increased the risk of RSV diagnosis, children with black race had 12% greater odds of RSV compared with white children (95% CI: 0.81, 1.56) (Table 2). These child characteristics were imprecise. Maternal characteristics were also not statistically significant, but regression results observed children with mothers < 20 years of age at delivery (OR: 1.15), multiparous mothers (OR: 1.20–1.27), and mothers who smoked (OR: 1.34) to have an increased the risk of RSV diagnosis (Table 2).

Community-level characteristics also rendered imprecise results, with children residing in a census tract with a high proportion of less than college education to have an 11% increased risk of RSV diagnosis (95% CI: 0.87, 1.42) (Table 2).

Our multivariable regression estimates also revealed that higher children’s age and private insurance were statistically significant protective factors for influenza diagnosis (Table 2). Younger children were at a decreased risk of influenza diagnosis. Compared with children aged 5 and older, children under 6 months of age had 90% reduced odds of influenza (95% CI: 0.06, 0.17). Children with public or self-pay insurance type had an increased risk for influenza diagnosis compared with children who had private insurance type. Those with self-pay had 2.12 times the odds of influenza diagnosis compared to those with private insurance (95% CI: 1.13, 4.00). Although estimates of all other predictors were imprecise, increased odds of influenza diagnosis was seen for children with high birthweight, of non-White race, with younger mothers (< 20 years), and with multiparous mothers (Table 2).

Stepwise regression analyses considered all potential predictors for RSV and influenza. When considering RSV, age (p < 0.0001), respiratory season (p = 0.0057), and maternal perinatal smoking (p = 0.0936) were included in the final model. When considering influenza, age (p < 0.0001), respiratory season (p < 0.0001), insurance (p = 0.0049), and infant feeding type (p = 0.0090) were included in the final model.

Results of multiple imputation analyses were more precise but overall risk estimates were similar and did not impact interpretations of the findings (results not shown). Sensitivity analyses examining the predictors of influenza diagnosis among vaccinated children rendered similar results to the main analysis. A total of 266 children had a vaccination reported in their medical records, among them 43 (16%) children had a positive test and 223 had a negative test (Table 2). Higher children’s age remained a protective factor for influenza diagnosis (Table 2). Although imprecise, high birthweight, non-white race, and having multiparous mothers remained positively associated with risk of influenza diagnosis (Table 3). The main differences between the sensitivity analysis and the main analysis for influenza were with sex, mothers’ age, and insurance type. Having young mothers and non-private insurance no longer increased the risk for influenza diagnosis but results were not statistically significant. An imprecise inverse association was observed for female sex and influenza diagnosis, OR: 95% CI: 0.61 (0.31, 1.92). Overall, results of influenza risk among vaccinated children were comparable with the full sample.

## Discussion

In the present work, age was the most influential predictor for RSV and influenza diagnosis. After consideration of all other predictors, children aged 0.5–1 year of age had the highest risk of RSV diagnosis, compared with children five years of age or older. RSV is common among young children in the US, up to 70% of children acquiring infection in their first year of life [4]. The present study was able to observe consistent results among children with a RSV diagnosis of all severity types, as opposed to focusing on children who are hospitalized with severe RSV disease. The average age of children who tested positive for influenza was 5.15 years. This is consistent with national estimates demonstrating influenza infection is greatest among children aged 5–14 years of age while, influenza-related complications, hospitalization, and mortality are greatest among children < 5 years of age [19].

In addition to age, insurance status was positively associated with influenza diagnosis. This was also confirmed in the stepwise regression model (p = 0.0049). A study of children with H1N1 influenza infection in Texas during the 2009 influenza pandemic observed that children with public or no insurance were less likely than those with private insurance to be hospitalized (p < 0.01) [20]. However, among non-hospitalized children, there was a doubling in the number of cases in influenza among those with public insurance as compared with private insurance [20]. A surveillance study of influenza from 2009–2013 observed individuals with no insurance to have 3.8 times the risk of influenza diagnosis compared to those with public insurance (95% CI: 1.4, 10.4) [21].

In the present study, all other associations were imprecise although some predictors showed relatively high positive associations with RSV and influenza, including race, birthweight, maternal age, parity, and maternal smoking. Previous studies that have considered similar predictors for RSV-associated hospitalizations have observed that age, race, and insurance are important determinants of RSV hospitalization [1]. In addition, associations with comorbid conditions indicative of prematurity and low birthweight have been observed [1,22–25]. After adjustment for gestational age, a study among children born in Ontario between 2012–2018 found that children < 2000 g at birth had a 57% increased risk of RSV-associated hospitalization compared with children of typical birthweight (95% CI: 1.24, 1.99) [24]. Similarly, in the present study we observed children with low birthweight (< 2500 grams) to have a 14% increased risk of RSV diagnosis compared to those of typical birthweight, although results were imprecise. Epidemiologic evidence has observed low birthweight to be associated with shorter telomere length, lower CD3 concentrations, and elevated C-reactive protein concentrations among preschool children, suggesting reduced immunocompetence and potential vulnerability to infections in childhood [26].

In the present study, there was an observed spike in positive influenza tests during the 2018–2019 seasons (Figure 2). According to the Centers for Disease Control and Prevention (CDC), this season did not exhibit an exceptionally high burden for this age group and so this observed trend is not consistent with that of the US population during this time [27]. Given RSV did not show the same trend, this may be related to influenza testing guidelines leading to increases in testing in the hospital and other affiliated clinics where these children were evaluated. This is also consistent with national estimates of increased influenza testing starting in 2017 [27].

The present work was among a specific sample of children who resided in Rochester, NY. The sample was restricted to children who were born at the Strong Memorial hospital, this led to a restriction in residential location of these children due to linkage to SPDS data. This resulted in a more homogeneous group of children with relation to socio-economic and community-level characteristics, evidenced by the null associations observed for the community- level socio-economic factors. Among our sample, 50% of participants were of non-White race, > 10% of Hispanic ethnicity, and > 60% had public insurance. Since the sample restriction was related to location of birth, our missing data were not missing at random. Due to this systematic missingness, multiple imputations were not an appropriate method to use.

Sensitivity analyses examining the predictors of influenza diagnosis among vaccinated children observed similar results to the full sample, albeit with less precision due to a further reduction in sample size. Influenza vaccine coverage in the US is associated with race, ethnicity, and SES factors [28,29]. The restrictions in our study sample may have led to the different observations in the present study. Despite this limitation, this study further supported evidence of sex differences. In our analysis females appeared to have a 39% reduced risk of influenza diagnosis, as compared with males. This is consistent with studies among children and adolescents which have observed sex differences among vaccine responses [30–33].

The present study has three key strengths. Many demographic, maternal, and socio-economic predictors were considered in the present analysis. This is one of few studies which have included a comprehensive list of non-clinical determinants for RSV and influenza diagnosis among children as most studies have focused on clinical predictors. In addition, this work investigated laboratory confirmed RSV and influenza diagnosis of all severities in the same study sample. Most literature examines these conditions separately with a focus on hospitalized or severe cases of disease. Although the restricted sample may be considered a limitation due to generalizability, discussed below, it can also be considered strength as this study was able to examine a low-income, underrepresented, urban sample of children. And lastly, RSV and influenza data were available over seven respiratory seasons and so trends of diagnosis could be observed.

The present study also has limitations: 1) Lack of clinical predictors, 2) Specific study sample, and 3) No influenza vaccine information. Most other studies examining predictors of RSV and influenza diagnosis among children have only considered clinical predictors. These predictors include underlying medical conditions, symptoms, and other genetic and immune- related factors [7,10,20,22,34–36]. These predictors have been relatively well-established, however, comprehensive examinations of the demographic, socio-economic, and maternal factors around these diagnoses in childhood are lacking. As previously described, given non-random missingness to the study sample, a restricted sample of children residing in the City of Rochester, NY was investigated. Although this sample is not generalizable to the larger United States population it is likely representative of other pediatric populations with a higher proportion of Black race, lower-income and SES households, and residence in an urban setting. Lastly, despite influenza vaccine information not being available for all study subjects, a sensitivity analysis among children with a reported vaccination rendered similar results with the full sample.

In conclusion, while considering multiple demographic, maternal, and community-level socio-economic predictors of RSV and influenza diagnosis among children, age was observed as the strongest predictor of both infections. Future work should examine these predictors in addition to clinical predictors among community cases of RSV and influenza, including severe and non-severe cases.

## Figures and Tables

**Table 1:** Descriptive associations between demographic, maternal, community-level characteristics and RSV and influenza diagnosis (N = 1808).

	RSV			Influenza		
	Positive n (%)	Negative n (%)	OR (95%CI)	Positive n (%)	Negative n (%)	OR (95%CI)
**Child Characteristics**						
**Age**						
< 0.5	130 (30)	261 (19)	15.26 (8.61, 27.05)	17 (6)	374 (25)	0.09 (0.05, 0.15)
0.5–1	79 (18)	149 (11)	16.25 (8.93, 29.55)	21 (7)	207 (14)	0.20 (0.12, 0.32)
1–1.5	66 (15)	150 (11)	13.48 (7.36, 24.71)	14 (5)	202 (13)	0.14 (0.08, 0.24)
1.5–2	44 (10)	105 (8)	12.84 (6.78, 24.31)	19 (6)	130 (9)	0.30 (0.17, 0.38)
2–3	44 (10)	110 (8)	12.26 (6.48, 23.17)	28 (9)	126 (8)	0.43 (0.28, 0.68)
3–4	29 (7)	102 (7)	8.71 (4.44, 17.08)	23 (8)	108 (7)	0.42 (0.26, 0.68)
4–5	26 (6)	70 (5)	11.38 (5.67, 22.85)	24 (8)	72 (5)	0.65 (0.39, 1.08)
≥ 5	14 (3)	429 (31)	1.00	150 (51)	293 (19)	1.00
**Age (years)** ^a^	1.05 (0.42–2.06)		0.75 (0.71, 0.79)	5.15 (2.22–7.94)		1.20 (1.16, 1.24)
**Sex**						
Male	242 (56)	728 (53)	1.00	160 (54)	810 (54)	1.00
Female	190 (44)	648 (47)	0.88 (0.71, 1.10)	136 (46)	702 (46)	0.98 (0.76, 1.26)
**Birthweight (grams)**						
< 2,500	135 (31)	352 (26)	1.36 (1.07, 1.73)	54 (18)	433 (29)	0.56 (0.41, 0.77)
2,500–4,000 (typical)	264 (61)	934 (68)	1.00	219 (74)	979 (65)	1.00
≥ 4,000	33 (8)	90 (7)	1.30 (0.85, 1.98)	23 (8)	100 (7)	1.03 (0.64, 1.66)
**Race**						
White	213 (49)	707 (51)	1.00	135 (46)	785 (52)	1.00
Black	156 (36)	496 (36)	1.04 (0.82, 1.32)	114 (39)	538 (36)	1.23 (0.94, 1.62)
Other	63 (15)	173 (13)	1.21 (0.87, 1.68)	47 (16)	189 (13)	1.45 (1.00, 2.09)
**Ethnicity**						
Non-Hispanic	383 (89)	1213 (88)	1.00	253 (85)	1343 (89)	1.00
Hispanic	49 (11)	163 (12)	0.95 (0.68, 1.34)	43 (15)	169 (11)	1.35 (0.94, 1.94)
**Insurance Type**						
Private	146 (33)	458 (33)	1.00	88 (30)	516 (34)	1.00
Public	267 (62)	839 (61)	1.00 (0.79, 1.26)	185 (63)	921 (61)	1.18 (0.89, 1.55)
Self-Pay	19 (4)	79 (6)	0.76 (0.44, 1.29)	23 (8)	75 (5)	1.80 (1.07, 3.02)
**Respiratory Season**						
2012–2013	63 (15)	170 (12)	1.00	40 (14)	193 (13)	1.00
2013–2014	55 (13)	96 (7)	1.55 (1.00, 2.40)	14 (5)	137 (9)	0.49 (0.26, 0.94)
2014–2015	58 (13)	140 (10)	1.12 (0.73, 1.70)	36 (12)	162 (11)	1.07 (0.65, 1.76)
2015–2016	55 (13)	164 (12)	0.91 (0.59, 1.38)	26 (9)	193 (13)	0.65 (0.38, 1.11)
2016–2017	67 (16)	173 (13)	1.05 (0.70, 1.57)	39 (13)	201 (13)	0.94 (0.58, 1.52)
2017–2018	79 (18)	349 (25)	0.61 (0.42, 0.89)	42 (14)	386 (26)	0.53 (0.33, 0.84)
2018–2019	55 (13)	284 (21)	0.53 (0.35, 0.79)	99 (34)	240 (16)	1.99 (1.32, 3.01)
**Maternal Characteristics**						
**Age**						
< 20	30 (7)	100 (7)	0.93 (0.58, 1.48)	31 (10)	99 (7)	1.46 (0.90, 2.35)
20–24.9	98 (23)	303 (22)	1.00	71 (24)	330 (22)	1.00
25–29.9	103 (24)	337 (25)	0.95 (0.69, 1.30)	68 (23)	372 (25)	0.85 (0.59, 1.22)
30–34.5	122 (28)	384 (28)	0.98 (0.72, 1.33)	75 (25)	431 (29)	0.81 (0.57, 1.15)
≥ 35	79 (18)	252 (18)	0.97 (0.69, 1.36)	51 (17)	280 (19)	0.85 (0.57, 1.26)
**Parity**						
0	155 (36)	568 (41)	1.00	117 (40)	606 (40)	1.00
1	137 (32)	414 (30)	1.21 (0.93, 1.58)	85 (29)	466 (31)	0.95 (0.70, 1.28)
≥ 2	140 (32)	394 (29)	1.32 (1.00, 1.69)	94 (32)	440 (29)	1.11 (0.82, 1.45)
**Feeding Type at Delivery**						
Breastmilk Only	129 (30)	500 (36)	1.00	128 (43)	501 (33)	1.00
Formula and Breastmilk	87 (20)	269 (20)	1.25 (0.92, 1.71)	59 (20)	297 (20)	0.78 (0.55, 1.09)
Formula Only	164 (38)	498 (36)	1.28 (0.98, 1.66)	102 (34)	560 (37)	0.71 (0.54, 0.95)
Neither	52 (12)	109 (8)	1.85 (1.26, 2.71)	7 (2)	154 (10)	0.18 (0.08, 0.39)
**Any Perinatal Smoking**						
No	353 (82)	1166 (85)	1.00	244 (82)	1275 (84)	1.00
Yes	79 (18)	210 (15)	1.24 (0.94, 1.65)	52 (18)	237 (16)	1.15 (0.82, 1.60)
**Community-Level**						
**High Proportion Unemployment**						
No	263 (61)	828 (60)	1.00	177 (60)	914 (60)	1.00
Yes	169 (39)	548 (40)	0.97 (0.78, 1.21)	119 (40)	598 (40)	1.03 (0.80, 1.33)
**High proportion with less than bachelor’s degree**						
No	193 (45)	681 (49)	1.00	146 (49)	728 (48)	1.00
Yes	239 (55)	695 (51)	1.21 (0.98,1.51)	150 (51)	784 (52)	0.95 (0.74, 1.22)
**High proportion living below poverty**						
No	279 (65)	883 (64)	1.00	191 (65)	971 (64)	1.00
Yes	153 (35)	493 (36)	0.98 (0.78, 1.23)	105 (35)	541 (36)	0.99 (0.76, 1.28)

a Median (IQR) for age in years among cases

**Table 2:** The association between various demographic, maternal, and community- level predictors and RSV and influenza diagnosis (N = 1,808).

Predictors	RSV OR (95% CI)	Influenza OR (95% CI)
**Child Age**		
< 0.5	14.14 (7.83, 25.56)	0.10 (0.06, 0.17)
0.5–1	15.97 (8.64,29.49)	0.22 (0.13, 0.37)
1–1.5	13.77 (7.43, 25.52)	0.14 (0.08, 0.25)
1.5–2	12.95 (6.77, 24.75)	0.29 (0.17, 0.50)
2–3	13.28 (6.95, 25.39)	0.44 (0.27, 0.71)
3–4	9.00 (4.56, 17.76)	0.40 (0.24, 0.67)
4–5	12.12 (5.99, 24.55)	0.62 (0.37, 1.04)
≥ 5	1.00	1.00
**Sex**		
Male	1.00	1.00
Female	0.90 (0.72, 1.14)	0.93 (0.71, 1.23)
**Ethnicity**		
Non-Hispanic	1.00	1.00
Hispanic	1.09 (0.70, 1.69)	0.90 (0.57, 1.44)
**Birthweight (grams)**		
< 2,500	1.14 (0.85, 1.52)	0.76 (0.53, 1.09)
2,500–4,000 (typical)	1.00	1.00
≥ 4,000	1.25 (0.79, 1.97)	1.14 (0.68, 1.92)
**Race**		
White	1.00	1.00
Black	1.12 (0.81, 1.56)	1.25 (0.85, 1.83)
Other	1.25 (0.84, 1.87)	1.42 (0.90, 2.25)
**Insurance Type**		
Private	1.00	1.00
Public	0.81 (0.59, 1.11)	1.26 (0.88, 1.83)
Self-Pay	0.57 (0.31, 1.05)	2.12 (1.13, 4.00)
**Respiratory Season**		
2012–2013	1.00	1.00
2013–2014	1.59 (0.99, 2.54)	0.52 (0.26, 1.05)
2014–2015	1.28 (0.81, 2.02)	1.02 (0.59, 1.76)
2015–2016	1.00 (0.64, 1.56)	0.62 (0.35, 1.10)
2016–2017	1.10 (0.72, 1.71)	1.04 (0.61, 1.77)
2017–2018	0.77 (0.52, 1.16)	0.39 (0.24, 0.65)
2018–2019	0.81 (0.51, 1.26)	1.28 (0.81, 2.04)
**Mom Age**		
< 20	1.15 (0.69, 1.92)	1.43 (0.83, 2.48)
20–24.9	1.00	1.00
25–29.9	0.83 (0.58, 1.18)	0.84 (0.55, 1.27)
30–34.5	0.85 (0.58, 1.24)	0.84 (0.55, 1.30)
≥ 35	0.81 (0.53, 1.23)	0.88 (0.54, 1.42)
Parity		
0	1.00	1.00
1	1.20 (0.90, 1.61)	1.17 (0.82, 1.66)
≥ 2	1.27 (0.93, 1.75)	1.41 (0.96, 2.06)
**Any perinatal smoking**		
No	1.00	1.00
Yes	1.34 (0.98, 1.85)	1.07 (0.74, 1.55)
**Feeding type at delivery**		
Breastmilk Only	1.00	1.00
Formula and Breastmilk	0.99 (0.69, 1.42)	0.73 (0.49, 1.09)
Formula Only	0.92 (0.68, 1.25)	0.92 (0.65, 1.29)
Neither	1.08 (0.68, 1.71)	0.30 (0.13, 0.71)
**High proportion with less than bachelor’s degree**		
No	1.00	1.00
Yes	1.11 (0.87, 1.42)	0.97 (0.72, 1.30)
High **proportion living below poverty**		
No	1.00	1.00
Yes	0.92 (0.65, 1.30)	0.83 (0.55, 1.24)
**High proportion unemployment**		
No	1.00	1.00
Yes	0.83 (0.61,1.14)	1.09 (0.76,1.57)

**Table 3:** The association between child, maternal, community-level characteristics, and influenza diagnosis among vaccinated children (N = 266).

	Influenza Positive N = 43 n (%)	Influenza Negative N = 223 n (%)	OR (95% CI)
**Child Characteristics**			
**Age**			
< 0.5	2 (5)	53 (24)	0.10 (0.02, 0.46)
0.5–1	2 (5)	27 (12)	0.20 (0.04, 0.92)
1–1.5	2 (5)	23 (10)	0.24 (0.05, 1.09)
1.5–2	3 (7)	13 (6)	0.63 (0.16, 2.42)
2–3	3 (7)	14 (6)	0.58 (0.15, 2.23)
3–4	4 (9)	17 (8)	0.64 (0.19, 2.12)
4–5	5 (12)	16 (7)	0.85 (0.28, 2.60)
≥ 5	22 (51)	60 (27)	1.00
**Sex**			
Male	27 (63)	113 (51)	1.00
Female	16 (37)	110 (49)	0.61 (0.31, 1.92)
**Birthweight (grams)**			
< 2,500	6 (14)	70 (32)	0.38 (0.15, 0.96)
2,500–4,000 (typical)	32 (74)	143 (64)	1.00
≥ 4,000	5 (12)	10 (4)	2.23 (0.72, 6.99)
**Race**			
White	20 (47)	104 (47)	1.00
Black	16 (37)	88 (40)	0.95 (0.46, 1.94)
Other	7 (16)	31 (14)	1.17 (0.45, 3.04)
**Ethnicity**			
Non-Hispanic	36 (84)	190 (85)	1.00
Hispanic	7 (16)	33 (15)	1.12 (0.46, 2.73)
**Insurance Type**			
Private	12 (28)	66 (30)	1.00
Public	29 (67)	145 (65)	1.10 (0.53, 2.29)
Self-Pay	2 (5)	12 (5)	0.92 (0.18, 4.63)
**Respiratory Season**			
2012–2013	6 (14)	26 (12)	1.00
2013–2014	0 (0)	19 (9)	< 0.001 (< 0.001, > 999.999)
2014–2015	6 (14)	26 (12)	1.00 (0.29, 3.51)
2015–2016	4 (9)	26 (12)	0.67 (0.17, 2.64)
2016–2017	6 (14)	36 (16)	0.72 (0.21, 2.49)
2017–2018	7 (16)	54 (24)	0.56 (0.17, 1.84)
2018–2019	14 (33)	36 (16)	1.69 (0.57, 4.97)
**Maternal Characteristics**			
**Age**			
< 20	2 (5)	10 (4)	1.07 (0.21, 5.49)
20–24.9	12 (28)	64 (29)	1.00
25–29.9	6 (14)	57 (26)	0.56 (0.20, 1.59)
30–34.5	15 (35)	53 (24)	1.51 (0.65, 3.50)
≥ 35	8 (19)	39 (17)	1.09 (0.41, 2.91)
**Parity**			
0	13 (30)	77 (35)	1.00
1	14 (33)	77 (35)	1.08 (0.48, 2.44)
≥ 2	16 (37)	69 (31)	1.37 (0.62, 3.06)
**Feeding type at delivery**			
Breastmilk Only	20 (47)	77 (35)	1.00
Formula and Breastmilk	10 (23)	49 (22)	0.79 (0.34, 1.82)
Formula Only	11 (26)	77 (35)	0.55 (0.25, 1.23)
Neither	2 (5)	20 (9)	0.39 (0.08, 1.79)
**Any perinatal smoking**			
No	37 (86)	181 (81)	1.00
Yes	6 (14)	42 (19)	0.70 (0.28, 1.76)
**Community-level**			
**High proportion unemployment**			
No	24 (56)	128 (57)	1.00
Yes	19 (44)	95 (43)	1.07 (0.55, 2.06)
**High proportion with less than bachelor’s degree**			
No	23 (53)	113 (51)	1.00
Yes	20 (47)	110 (49)	0.89 (0.46, 1.72)
**High proportion living below poverty**			
No	26 (60)	130 (58)	1.00
Yes	17 (40)	93 (42)	0.91 (0.47, 1.78)
